# Health Risk Assessment of Indoor Air Quality, Socioeconomic and House Characteristics on Respiratory Health among Women and Children of Tirupur, South India

**DOI:** 10.3390/ijerph14040429

**Published:** 2017-04-17

**Authors:** Krassi Rumchev, Yun Zhao, Jeffery Spickett

**Affiliations:** School of Public Health, Curtin University, Perth, WA 6148, Australia; y.zhao@curtin.edu.au (Y.Z.); j.spickett@curtin.edu.au (J.S.)

**Keywords:** indoor air quality, socio economic status (SES) house characteristics, particulates, developing countries, respiratory health

## Abstract

*Background:* Indoor air pollution is still considered as one of the leading causes of morbidity and mortality worldwide and especially in developing countries, including India. This study aims to assess social, housing, and indoor environmental factors associated with respiratory health among mothers and children. *Methods:* The study was conducted in the city of Tirupur, South India. We quantitatively assessed the indoor exposure to fine particulate matter (PM_2.5_) and carbon monoxide in relation to respiratory health among women and children. Information on health status, household characteristics and socioeconomic factors was collected using a modified standardised questionnaire. *Results:* This study demonstrates the significant health impact of housing and socioeconomic characteristics on the burden of respiratory illness among women and children in urban South India. Increased respiratory symptoms were recorded among women and children from low income households, and those who allowed smoking inside. The mean PM_2.5_ concentration measured in this study was 3.8 mg/m^3^ which exceeded the World Health Organization (WHO) 24 h guideline value of 0.025 mg/m^3^. *Conclusions:* This study is the first to our knowledge carried out in urban South India and the findings can be used for future intervention studies.

## 1. Introduction

The World Health Organization (WHO) has assessed the contribution of a range of risk factors to the burden of disease and revealed indoor air quality as the eight most important risk factor and responsible for 2.7% of the global burden of disease [1]. Poor indoor air quality is recognised as a significant risk factor for respiratory health and especially in lower income countries including India. Nowadays, people spend substantial proportion of their time indoors and may become exposed to elevated levels of air pollutants. The level of exposure to indoor air pollution is influenced by multiple factors, both macro-environmental and micro-environmental determinants. Micro-environmental determinants include housing characteristics, whereas macro-environmental determinants consist of demographic and socioeconomic factors [2]. Some of the main housing characteristics that may contribute to indoor air pollutants include type of fuel for cooking and heating, and occupants’ activities such as tobacco smoke. Tobacco smoke is considered as one of the most common indoor air pollutants which exposes non-smokers such as children and most mothers. Second-hand smoke consists of a mixture of more than 4000 chemicals, of which, more than 40 are identified carcinogens in vapour and particulate phases [3]. According to Besaratinia and colleagues [4], second-hand smoking can be more carcinogenic than mainstream smoke inhaled by active smokers. Combustion of fuels for cooking continues to be one of the most widespread and important sources of exposure to indoor air pollution and especially in developing countries where solid fuel is still in use. According to Smith [5], 52% of the world’s population uses solid fuels with some countries, including India, having a higher percentage of 74% compared to 16% in Central and Eastern Europe. Solid fuel can be biomass or coal and during combustion can release a complex mixture of air pollutants including inorganic gases such as CO, NO_2_, particulate matter (PM), organic compounds, hydrocarbons and free radicals [6]. Particulate matter (PM) is a widespread air pollutant, consisting of a mixture of solid and liquid particles and it is used as an indicator pollutant in many air quality studies [7]. PM is classified by aerodynamic diameter as size is a critical health determinant. Fine particulate matter (PM_2.5_) with an aerodynamic diameter less than 2.5 microns, have the greatest potential for harmful health effects with the ability to penetrate deep into the lungs where gas-exchange occurs [1]. There is considerable evidence to show that indoor air pollution (IAP) in developing countries is associated with increased health risks [8]. According to Smith and colleagues [7], it is estimated that 4%–6% of the total burden of disease in India can be attributed to IAP, however, the burden from poor IAP increases to 6%–9% for mothers and children under five since they spend most of the time indoors. Young children are considered more vulnerable than adults from exposure to air pollution as their immune system and lungs are not fully developed [9]. 

Despite the growing body of literature addressing the wide range of health impacts associated with poor indoor air quality, there is a limited number of studies from developing countries that investigated the health impact of socioeconomic and housing factors on mothers and children. The objective of this study was to assess social, housing, and indoor environmental factors associated with respiratory health among mothers and children in the city of Tirupur. This study is the first to our knowledge conducted in urban South India and can contribute with some important data for future intervention studies.

## 2. Materials and Methods

### 2.1. Study Population

Two hundred and fifty households living in small communities in the city of Tirupur located in Coimbatore District of Tamil Nadu State, South India were approached and 170 agreed to take part in the first stage of this study consisting of questionnaire survey. Of the 170 families, 80 consented for indoor air quality monitoring. Approval for the study was granted by the Curtin University Research Ethics Committee, Australia (Ref. No. 40.2006) and a signed informed consent was obtained from each participating household. 

### 2.2. Data Collection

Data collection comprised two stages. Stage one involved a standardized health and housing survey conducted through face-to-face interviews between researchers and mothers. Stage two involved indoor air quality monitoring. 

#### 2.2.1. Respiratory Health Survey

Information related to study participants’ demographic and housing characteristics, and health status was gathered using a survey, adopted from a standardized questionnaire on indoor air quality (IAQ) and health of the American Thoracic Society [10] and successfully used in other similar studies [11,12]. Some questions were modified to better reflect the local lifestyle and settings of the study area. The questionnaire was translated into the local language, Tamil, by a team of local medical and environmental health officers. Pre-test of the questionnaire was conducted among 10 families a few weeks prior to the survey to evaluate the questionnaires comprehensibility, sensitivity of questions and appropriateness of language. The questionnaire consisted of three sections and the aim of part one was to collect demographic data, including questions related to age and gender. Health questions were also asked including the experience of cough, wheeze, and breathlessness among mothers and children in the last 6 months. Section two gathered information related to household characteristics and occupants’ activities, with particular emphasis on cooking and indoor smoking. The final part of the survey asked questions related to socioeconomic status (SES) such as number of children, parents’ education and family income.

#### 2.2.2. Indoor Air Quality Assessment

The second stage of the study involved a quantitative assessment of indoor concentrations of CO and PM_2.5_. Of the 170 households who participated in the survey, 80 agreed to take part in the indoor air quality assessment. Measurements were conducted in the kitchen area for approximately 4 h including cooking time. The monitoring equipment was positioned at about 1 m above the ground. A DustTrak Aerosol Monitor model 8520 (TSI Inc., Shoreview, MN, USA), was used for monitoring of fine particles (PM_2.5_). This is a real-time monitor with the flow rate of 1.7 L/min. In addition to the regular annual factory calibration, the DustTrak was custom calibrated using the integral 37 mm filter, at some selected site locations, to determine the gravimetric concentration. The custom calibration factor was reused at all measurement sites. The levels of exposure to CO were estimated using Dräeger pump (Model 21/31) as successfully used in previous studies [11,12]. The limit of detection (LOD) for CO Dräeger tube was 2.47 mg/m^3^ and the detection range between LOD and 74 mg/m^3^ (±10%). Other indoor parameters such as temperature and humidity were monitored using Tiny tag-Ultra Data Loggers with built-in sensors.

#### 2.2.3. Statistical Analysis

In the current study, respiratory symptoms for mothers and children were the main outcomes and they were recoded as binary variables (0 = had no symptoms, 1 = had symptoms). Upper respiratory symptoms were defined as runny nose and coughs, whereas lower respiratory symptoms were defined as wheeze, shortness of breath, asthma, chest pain, and bronchitis.

Independent/exposure variables, including demographic factors, household kitchen characteristics, domestic environment conditions (smoking: family member and visitors) and indoor air pollution variables were recoded into binary or categorical variables with more than two levels. In this study, the indicators for socioeconomic status included family income, maternal education and occupation of mothers and fathers.

Descriptive statistics were generated for the outcome and independent variables. Bivariate analyses, including cross-tabulations and chi-squares tests, were performed for assessing unadjusted associations between the independent/exposure variables and respiratory symptoms as outcome variables. As the current study is cross-sectional with common occurred health outcomes, a multivariable Poisson regression (MPR) analysis with robust variance estimation was considered as appropriate [13] to examine the adjusted association (1) between respiratory symptoms and household characteristics, kitchen activities and domestic environment conditions, controlling for confounders (demographic factors, socioeconomic status and smoking), and (2) between respiratory symptoms and indoor air pollution is depicted by PM_2.5_ and CO, controlling for aforementioned confounders, and indoor temperature and relative humidity. The multivariable modelling was carried out at two stages, namely a complete and reduced modelling. In the complete modelling, all variables describing socioeconomic status, household characteristics, and domestic environment conditions were included. In the supplementary reduced modelling, only those variables found to be significantly associated with the health outcomes in the univariate analysis with *p* < 0.1 were included (reported in the paper). Backward elimination approach was applied to get the parsimonious final model. Crude and adjusted prevalence ratios and their 95% confidence interval (CI) are reported. Crude prevalence ratio (CPR) is reported when associations are significant but not adjusted for confounders. Only variables still significantly (*p* < 0.05 or *p* < 0.1) associated with mothers’ and children’s respiratory symptoms after controlling for confounders in the final model are reported as adjusted prevalence ratios (APR). 

In addition, the association between household characteristics, socioeconomic status and indoor air quality was examined. Independent *t*-test or one-way ANOVA were used to investigate the difference between groups. Multivariable linear regression (MLR) analysis with a backward elimination approach was conducted to examine the effect of household characteristics, kitchen activities and domestic environment conditions on indoor air pollution concentrations, controlling for socioeconomic status. 

Statistical analysis was performed using the IBM SPSS Version 21.0 for windows (IBM SPSS Statistics for Windows, Version 21.0. IBM Corp., Armonk, NY, USA). The critical level of significance of 5% for all statistical tests (two-tailed) was used in this study. 

## 3. Results

### 3.1. Study Population—Demographic Characteristics and Socioeconomic Status

A total of 170 households signed a consent form to participate in the questionnaire survey. The age of mothers ranged between 19 and 45 years, and for children between 1 and 15 years. More than half of the families (52%) had two or three children, 23% had only one child, and 27% reported more than three children. Majority of mothers (98%) had primary education (Year 7), 2% had secondary education (Years 8–12), and only one mother reported having University degree (BA degree). One hundred and thirty nine houses (82%) earned less than 1000 Indian rupee (INR) (<A$31) per month and 18% (n = 31) earned between 1000 and 3000 INR (<A$94) per month (Table 1). The income cut-off for poverty was 1000 INR.

### 3.2. Household Characteristics

Most of the households had either one or two rooms (n = 141, 83%) (Table 2). 

Seventy-four houses (43%) had mud and 96 (56%) cement for floor covering and no households reported to have carpet. There were 70% of the houses (n = 119) with a kitchen or stove located inside the house (with or without partitions). Most of the kitchen roofs were covered with tiles (71%, n = 120), followed by thatch (made of coconut leaves) (24%, n = 40), asbestos (7%, n = 4), and iron sheets (2%, n = 3). Biomass was the most prevalent fuel used for cooking 125 (74%, n = 125) followed by liquid petroleum gas (LPG) 28 (16%, n = 28) and only 17 (10%) used kerosene. No families reported the use of coal or electricity for cooking. According to the survey reports, none of the mothers ever smoked but almost half of the fathers 78 (46%) reported smoking. In addition, thirty-eight percent of the households allowed their guests to smoke inside the house.

### 3.3. Indoor Air Quality 

Among the 170 households participating in the survey, 80 families agreed to take part in the indoor air quality monitoring including exposure to PM_2.5_ and CO. The mean and median concentrations of PM_2.5_ were 3.80 mg/m^3^ and 1.18 mg/m^3^, respectively, with a range from 0.04 mg/m^3^ to 83.84 mg/m^3^. This result demonstrates that all households were exposed to higher than the WHO guideline value of 0.025 mg/m^3^. In fact, the majority of households (80%) were exposed to ten times higher PM concentrations than the guideline value which is in contrast with the findings for CO. Eighty-three percent of houses (n = 66) met the WHO 8-h CO guideline values of 10 mg/m^3^ [14] and the highest CO concentration was 50 mg/m^3^. The median and mean concentrations of carbon monoxide (CO) were <LOD (2.47 mg/m^3^) and 4.63 mg/m^3^, respectively, with the range from <(LOD) to 50 mg/m^3^. The mean temperature measured in the households was 30 °C (24 °C–43 °C) and the mean relative humidity was 52% (33%–82%) and both slightly exceeded the ASHRAE recommended guideline values for temperature (21 °C–26 °C) and relative humidity (30%–70%) [15].

### 3.4. Associations between Household Characteristics and Indoor Concentrations of PM_2.5_ and CO

The univariate analysis through independent sample t test showed that households who used mainly biomass for cooking were exposed to significantly (*p* < 0.01) higher median PM_2.5_ concentration of 1.32 mg/m^3^ (IQR: 2.34) compared with 0.16 mg/m^3^ (IQR: 0.41) for those who used LPG/others; households with tiled kitchen roof had a significantly lower (*p* = 0.037) median concentration value of PM_2.5_ (0.875 mg/m^3^, IQR = 1.98) compared to those with a thatch/asbestos/iron kitchen roof (1.415 mg/m^3^, IQR = 4.29). Women and children who lived in low income households were also exposed to significantly (*p* < 0.05) higher PM_2.5_ concentrations of 1.30 mg/m^3^ (IQR: 2.53) compared to those with a higher income (0.51 mg/m^3^; IQR: 0.5). Significantly (*p* < 0.05) higher PM concentration of 1.36 mg/m^3^ (IQR: 2.88) was measured when mothers kept windows closed while cooking when compared with 0.45 mg/m^3^ (IQR: 2.53) measured in households where windows were opened during cooking activities. Smoking in the house also affected the particle concentrations as children and women who lived in a smoke free house were exposed to significantly (*p* < 0.05) lower PM_2.5_ concentrations (1.12 mg/m^3^; IQR: 1.90) compared to those who lived in houses where smoking was allowed (2 mg/m^3^; IQR: 2.62). The MLR analysis further confirmed the significant relationship between type of cooking fuel and PM_2.5_ concentration, indicating that using firewood for cooking significantly (*p* = 0.006) increased the PM_2.5_ concentration by 1.368 mg/m^3^. With regard to CO exposure levels, almost half of the households, 46 (57%), had CO concentrations below the LOD.

### 3.5. Respiratory Health among Women and Children 

All mothers reported at least one respiratory symptom and the most common respiratory symptom was wheeze (53%), followed by cough (43%). Cough was reported among 34% of the children, followed by runny nose (23%) and wheeze (17%) (Table 3).

### 3.6. Socioeconomic Status, Household Characteristics and Occupant Activities and the Associated Respiratory Health among Women

In this study, the SES characteristic that made a significant health impact on women was the household income. According to the Poisson regression analysis, women who lived in households with income less than 1000 INR were more likely to have higher prevalence of cough, wheeze, shortness of breath and chest pain. House characteristics that made a significant contribution to respiratory symptoms included smoking inside, biomass fuel, time spent cooking, source of lighting, floor and wall coverings (Table 4).

### 3.7. Socioeconomic Status, Household Characteristics, Occupant Activities and Associated Respiratory Health among Children 

Low income and smoking inside the house had adverse health impacts on children which is consistent with the findings of their mothers. Children who lived in smoking households were almost five times more likely to experience hay fever and two times more likely to have cough compared to those who lived in a smoke free house. Kitchen materials and place for cooking also had a significant effect on children’s respiratory symptoms. During cooking, those children who stayed near mothers were likely to experience more respiratory symptoms and for every extra hour spent in the kitchen, the likelihood of having hay fever increased almost twice. More people in the kitchen also contributed to the increased prevalence of runny nose, hay fever, and eczema among children (Table 5). 

## 4. Discussion

In this study, we found that socioeconomic and housing characteristics had a significant association with respiratory health among mothers and children. Increased respiratory illnesses in both women and children were recorded in low income households and those that allowed smoking inside the house. Higher prevalence of respiratory symptoms was also reported among families who lived in mud houses compared with those in stone houses and this is consistent with a study conducted in Nepal [16]. This is probably because people who live in mud houses are considered generally poor. Exposure to biomass fuel, time spent in the kitchen, cooking place, closed windows and doors during cooking significantly increased the prevalence of respiratory symptoms among mothers and similar results are reported elsewhere [17,18,19].

In this study, we quantitatively assessed exposure to fine particulate matter and carbon monoxide in the participating households. There is a limited number of quantitative exposure assessment studies conducted in India and yet, the current study measured high mean PM_2.5_ concentration (3.80 mg/m^3^) which is consistent with the findings of previous studies conducted in other parts of India. In North India, Ansari [20] and Saksena [21] reported high mean PM_2.5_ levels (2.38 mg/m^3^ and 4.50 mg/m^3^, respectively). In the northwest of India, Smith [22] and Ingale [23], also recorded very high exposure levels to particulate air pollution, 6.80 mg/m^3^ and 6.28 mg/m^3^, respectively. In the current study, however, the mean PM concentration was lower than the measured exposure levels in North and Western India which can be explained by the different culture and climate conditions in South India. Tamil Nadu which is located in South India, and where the current study was carried out, has moderate to hot temperatures throughout the year and families are more likely to keep doors (84%) and windows (46%) open during cooking which is in contrast with North India where the cold weather remains longer. In addition, the design of houses and duration of cooking also differ between different parts of India and women in South India are likely to cook for less time compared with those who live in the northern parts of India [6]. In addition, the current study was conducted in urban South India and we measured higher mean particle concentration (3.80 mg/m^3^) compared with those measured in a similar study but conducted in rural South India (1.57 mg/m^3^) [24].

The results, reported in our study and other similar studies in India, are consistent with the findings from other countries. In a study conducted in Pakistan [25], the average exposure level of particulate air pollution was 8.55 mg/m^3^ and in Costa Rica [26] the maximum measured PM concentration was 18.9 mg/m^3^. Similar results are reported elsewhere [11,12,27]. With regard to carbon monoxide, Shrestha and colleagues [16] measured exposure levels to CO in Nepalese households that were well below the WHO recommended guideline value and this is consistent with findings of our study. In the current study, only 17% of participating households were exposed to higher concentrations than the WHO guideline value. 

This study did not establish an association between exposure to PM_2.5_ and CO, and respiratory symptoms among mothers and children. Bruce [28] reported that most studies from developing countries failed to demonstrate a significant association between indoor air pollutants and respiratory illnesses due to methodological limitations, including the small sample size which we believe is the case in the current study. We also recognise that other air pollutants including nitrogen oxides (NOx) are not measured in the current study, and could have affected the association between respiratory symptoms and indoor air pollution. In this study, the indoor air quality was monitored for 4 h which is acknowledged as a limitation considering the 24 h guideline value for PM_2.5_ and the 8 h guideline value for CO. Another study limitation is related to the diagnosis of asthma and bronchitis which may vary from country to country. In order to minimise potential misdiagnosis, we asked local trained medical professionals to assist with the interview process of the study population.

## 5. Conclusions

This study found that housing and socioeconomic characteristics have a significant health impact on women and children from India. Although there are other similar studies conducted in India, this study is the first to our knowledge carried out in urban South India and thus the findings can be used for future intervention studies. Intervention studies have proved that indoor air pollution levels can be lowered significantly by using different intervention strategies including improved stoves and improved ventilation such as installing chimneys, smoke hoods and enlarged windows. Behaviour changes through education may also play a role in reducing exposure to tobacco smoke which can reduce the prevalence of respiratory symptoms. Keeping children away during cooking can significantly decrease their exposure to health-damaging pollutants and therefore can reduce respiratory morbidity among them [8].

## Figures and Tables

**Table 1:** Demographic and socioeconomic characteristics of study population.

Household (n = 170)	Mean ± SD *	n (%)
**Mother (n = 170)**		
**Age (years)**	30.13 ± 5.99	
**Marital status** Married Widowed		162 (95.3) 8 (4.7)
**Education** Low High		165 (97.1) 5 (2.9)
**Occupation** Home Employed		80 (47.1) 90 (52.9)
**Family income** Low (<1000 rupees) High (1000< ~ 3000 rupees)		139 (81.8) 31 (18.2)
**Own the land** No Yes		132 (77.6) 38 (22.4)
**Own the house** No Yes		38 (22.4) 132 (77.6)
**Number of children aged <1 to 15 years** 0 1 2 3 4<		24 (14.1) 48 (28.2) 55 (32.4) 28 (16.5) 14 (8.9)
**Children (n = 308)**		
**Age (years)** <1–5 6–10 11–15		133 (43.2) 120 (39.0) 55 (17.8)

* SD = “Standard Deviation”.

**Table 2:** Household and kitchen characteristics (n = 170).

Characteristics	Mean ± SD	n (%)
**House**		
**Number of rooms** 1 2 3 4<	1.8 ± 1.0	76 (44.7) 65 (38.2) 18 (10.6) 11 (6.4)
**Number of people living in the house** <3 3 4 5 6 7<	4.5 ± 1.5	12 (7.1) 25 (14.7) 52 (30.6) 42 (24.7) 26 (15.3) 13 (7.7)
**Smoking at house (family member)** No Yes		92 (54.1) 78 (45.9)
**Smoking at house (visitors)** No Yes		132 (77.6) 38 (22.4)
**Kitchen**		
**Floor material** Clay/mud Cement		74 (43.5) 96 (56.5)
**Wall material** Thach/mud Stone/brick		60 (35.3) 110 (64.7)
**Roof material** Thach/asbestos Tile/iron		50 (29.4) 120 (70.6)
**Source of lighting used** Lanterns/gas Electricity		18 (10.6) 152 (89.4)
**Fuel used for cooking** Biomass LPG */other		125 (73.5) 45 (26.5)
**Stove** Enclosed chamber Open combustion		119 (70) 51 (30)
**Cooking place** Inside Outside		118 (69.8) 51 (30.2)
**Windows in kitchen** No Yes		80 (47.1) 90 (52.9)
**Open windows during cooking** No Yes		92 (54.1) 78 (45.9)
**Open door during cooking** No Yes		27 (15.9) 143 (84.1)
**Number of people in kitchens during cooking**	2.1 ± 1.5	
**Time spent for cooking (h)**	2.8 ± 0.7	

Note: * Liquid Petroleum Gas (LPG).

**Table 3:** Prevalence of respiratory symptoms among mothers and children.

Classification	Respiratory Symptoms in Mothers		Respiratory Symptoms in Children	
	Symptoms	n (%)	Symptoms	n (%)
**Upper** respiratory symptoms	**Cough**		**Cough**	
	No	97 (57.1)	No	113 (66.5)
	Yes	73 (42.9)	Yes	57 (33.5)
			**Runny nose**	
			No	131 (77.1)
			Yes	39 (22.9)
			**Had both runny nose and cough**	
			No	144 (84.7)
			Yes	26 (15.3)
**Lower** respiratory symptoms	**Wheeze**		**Wheeze**	
	No	79 (46.5)	No	141 (82.9)
	Yes	91 (53.5)	Yes	29 (17.1)
	**Shortness of breath**			
	No	129 (75.9)		
	Yes	41 (24.1)		
	**Ever had asthma**		**Ever had asthma**	
	No	169 (99.4)	No	167 (98.2)
	Yes	1 (0.6)	Yes	3 (1.8)
	**Ever had bronchitis**			
	No	146 (85.9)		
	Yes	24 (14.1)		
	**Pain in the chest**			
	No	109 (64.1)		
	Yes	61 (35.9)		
	**Had all lower respiratory symptoms**		**Had both runny nose and cough**	
	No	170 (100)	No	168 (98.8)
	Yes	0 (0)	Yes	2 (1.2)
**Other**	**Ever had pneumonia**			
	No	163 (95.9)		
	Yes	7 (4.1)		
	**Ever had emphysema**			
	No	0 (0)		
	Yes	170 (100)		
	**Ever had tuberculosis**			
	No	168 (98.8)		
	Yes	2 (1.2)		
	**Ever had hay fever**		**Ever had hay fever**	
	No	152 (89.4)	No	162 (95.3)
	Yes	18 (10.6)	Yes	8 (4.7)
			**Ever had itchy rash**	
			No	158 (92.9)
			Yes	12 (7.1)
			**Ever had eczema**	
			No	161 (94.7)
			Yes	9 (5.3)
			**Ever had tonsillectomy**	
			No	167 (98.2)
			Yes	3 (1.8)
	**Had all 4 ‘Other’ symptoms**		**Had all 4 ‘Other’ symptoms**	
	No	170 (100)	No	170 (100)
	Yes	0 (0)	Yes	0 (0)
**Overall**	**Had all 10 symptoms**		**Had all 8 symptoms**	
	No	170(100)	No	170 (100)
	Yes	0 (0)	Yes	0 (0)

**Table 4:** Association between socioeconomic status, household characteristics, occupant activities, and prevalence of respiratory symptoms among mothers.

Factor ^♌^	Symptom	CPR *	95% CI	APR ^§^	95% CI
**SES Indicators**					
**Family income^¶^** Low vs. High (ref)	Cough	2.490	(1.189, 5.214)		
	Wheeze	3.836	(1.701, 8.651)	3.521	(1.608, 7.692)
	Shortness of breath	4.349	(1.109, 17.075)		
	Pain in the chest	4.312	(1.445, 12.866)	4.065	(1.362, 12.048)
**Household Characteristics**					
**Source of lighting** Lanterns/gas vs. Electricity (ref)	Cough	1.661	(1.136, 2.429)		
	Wheeze	1.407	(1.016, 1.949)		
**Floor material** Clay/mud vs. Cement (ref)	Cough	1.572	(1.111, 2.225)		
	Wheeze	1.654	(1.248, 2.193)	1.352 ^✠^	(0.999, 1.829)
	Shortness of breath	2.502	(1.415, 4.423)	2.148	(1.216, 3.797)
**Wall material** Thach/mud vs. Stone/Brick (ref)	Cough	1.784	(1.277, 2.491)	1.742	(1.261, 2.405)
	Wheeze	1.87	(1.439, 2.441)	1.598	(1.197, 2.133)
	Pain in the chest			1.831	(1.184, 2.831)
**Fuel used for cooking** Biomass vs. LPG/other (ref)	Wheeze	1.824	(1.179, 2.822)		
	Shortness of breath	2.592	(1.085, 6.194)		
**Roof material** Thach/asbestos vs. Tile/Iron (ref)	Wheeze	0.740	(0.562, 0.973)	1.316	(1.024, 1.691)
	Pain in the chest			1.833	(1.101, 3.051)
**Cooking place** Inside vs. Outside (ref)	Cough	1.472	(1.046, 2.072)		
	Wheeze	1.615	(1.243, 2.098)		
	Ever had bronchitis	2.121	(1.003, 4.487)		
**Time spent for cooking (h)**	Shortness of breath	1.451	(1.197, 1.758)	1.443	(1.153, 1.806)
	Ever had asthma	2.510	(1.655, 3.807)	2.524	(1.684, 3.785)
	Ever had pneumonia	2.092	(1.299, 3.367)	2.219	(1.407, 3.499)
	Ever had hay fever	1.954	(1.608, 2.375)	1.820	(1.433, 2.432)
**Open window/s during cooking** No vs. Yes (ref)	Cough	1.628	(1.116, 2.375)		
	Wheeze	1.421	(1.054, 1.917)		
	Shortness of breath	1.826	(1.018, 3.276)		
**Open the door when cooking** No vs. Yes (ref)	Wheeze	1.791	(1.419, 2.262)		
	Pain in the chest	1.578	(1.024, 2.431)		
**Number people in kitchen when cooking**	Pain in the chest	1.142	(1.020, 1.279)	1.140	(1.011, 1.285)
**Other Activity**					
**Visitor smoking** Yes vs. No (ref)	Shortness of breath	1.696	(1.000, 2.877)		
	Ever had bronchitis	2.692	(1.251, 5.793)	2.339	(1.098,4.982)
	Ever had hay fever	8.077	(2.432, 26.826)	6.513	(2.006,21.142)

^♌^ Variables which were significant with *p* < 0.1 in univariate analyses were included initially and the backward elimination method was applied to achieve the final model. Only factors significantly (*p* < 0.05 or ^✠^ *p* < 0.1) associated with mothers’ symptoms were reported. * CPR = crude prevalence ratio, not adjusted for confounders. ^§^ APR = adjusted prevalence ratio, adjusted by age and other covariates/confounders. Low: low less than 1000 INR, high: between 1000–3000 INR. LPG = liquid petroleum gas. CI = Confidence Interval. ref = reference.

**Table 5:** Association between socioeconomic status, kitchen characteristics, occupant activities, and prevalence of children’s respiratory symptoms.

Factor ^♌^	Symptom	CPR *	95% CI	APR ^§^	95% CI
**SES**					
**Family income**^¶^ Low vs. High (ref)	Cough	4.014	(1.342, 12.005)	3.636	(1.167, 11.364)
	Wheeze	6.245 ^✠^	(0.883, 44.169)	8.197 ^✠^	(0.951, 71.429)
**Household Characteristics**					
**Floor material** Clay/mud vs. Cement (ref)	Cough	1.547	(1.012, 2.363)		
**Wall material** Thach/mud vs. Stone/Brick(ref)	Cough	1.899	(1.256, 2.870)	2.000	(1.339, 2.987)
	Wheeze	1.964	(1.018, 3.789)	1.993	(1.039, 3.825)
**Time spent for cooking (h)**	Ever had hay fever	1.599	(1.005, 2.420)		
	Ever had tonsillectomy	1.378	(1.089, 1.743)	1.362	(1.074, 1.727)
**Open windows during cooking** No vs. Yes (ref)	Cough	1.696	(1.070, 2.698)		
**Open door during cooking** No vs. Yes (ref)	Wheeze	2.018	(0.999, 4.075)		
**Number of people in kitchens during cooking**	Cough	1.124 ^✠^	(0.994, 1.270)	1.135 ^✠^	(0.993, 1.298)
	Runny nose	1.166	(1.000, 1.361)	1.152	(1.008, 1.316)
	Had both runny nose and cough	1.209	(0.993, 1.472)		
	Ever had hay fever	1.510	(1.109, 2.055)	1.565	(1.041, 2.415)
	Ever had itchy rash	1.436	(1.099, 1.877)	1.442 ^✠^	(0.986, 2.109)
	Ever had eczema	1.497	(1.120, 2.001)	1.487	(1.096, 2.017)
**Visitor smoking** Yes vs. No (ref)	Cough	2.350	(1.596, 3.460)	2.386	(1.652, 3.447)
	Wheeze	2.132	(1.100, 4.097)	2.288	(1.207, 4.337)
	Had both runny nose and cough	2.171	(1.075, 4.385)	2.089	(1.023, 4.264)
	Ever had hay fever	5.789	(1.449, 23.131)	6.562	(1.568, 27.453)

^♌^ Variables which were significant with *p* < 0.1 in univariate analyses were included initially and the backward elimination method was applied to achieve the final model. Only factors significantly (*p* <0.05 or ^✠^ *p* < 0.1) associated with mothers’ symptoms were reported. * CPR = crude prevalence ratio, not adjusted for confounders. ^§^ APR = adjusted prevalence ratio, adjusted by age and other covariates/confounders. Low: low less than 1000 INR, High: between 1000–3000 INR. SES = socio-economic status

## References

[1] WHO (2005). Air Quality Guidelines for Particulate Matter, Ozone, Nitrogen Dioxide and Sulfur Dioxide.

[2] Balakrishnan K., Nriagu J.O. (2011). Solid Fuel: Health Effects. Encyclopaedia of Environmental Health.

[3] United States Department of Health and Human Services The Health Consequences of Involuntary Exposure to Tobacco Smoke: A Report of the Surgeon General. https://www.surgeongeneral.gov/library/reports/secondhandsmoke/fullreport.pdf.

[4] Besaratinia A., Pfeifer G.P. (2008). Second-hand smoke and human lung cancer. Lancet Oncol..

[5] Smith K.R., Mehta S., Maeusezahl-Feuz M., Ezzatti M., Lopez A.D., Rodgers A., Murray C.J.L. (2004). Indoor air pollution from solid fuel use. Comarative Quantification of Health Risks: Global and Regional Burden of Disease Attributable to Selected Major Risk Factors.

[6] Bruce N., Perez-Padilla R., Albalak R. (2002). The Health Effects of In-Door Air Pollution Exposure in Developing Countries.

[7] Smith K., Samet J., Romieu I., Bruce N. (2000). Indoor air pollution in developing countries and acute respiratory infections in children. Thorax.

[8] World Health Organisation (WHO) (2014). Burden of Disease from Household Air Pollution for 2012. Summary of Results.

[9] Ashmore M.R., Dimitroulopoulou C. (2009). Personal exposure of children to air pollution. Atmos. Environ..

[10] American Thoracic Society (ATS) (1978). Recommended respiratory disease questionnaires for use with adults and children in epidemiological research. Am. Rev. Respir. Dis..

[11] Rumchev K., Spickett J., Brown H., Mkhweli B. (2007). Indoor air pollution from biomass combustion and respiratory symptoms of women and children in a Zimbabwean village. Indoor Air.

[12] Rumchev K., Win T., Bertolatti D., Satvinder D. (2015). Prevalence of respiratory symptoms among women and children in rural Myanmar-disease burden assessment attributable to household biomass smoke. Indoor Built Environ..

[13] Coutinho L., Scazufca M., Menezes P. (2008). Methods for estimating prevalence ratios in cross-sectional studies. Rev. Saude Publica.

[14] World Health Organization (WHO) (2010). Guidelines for Indoor Air Quality: Selected Pollutants.

[15] ANSI/ASHRAE Thermal Environmental Conditions for Human Occupancy. Atlanta: American Society of Heating, Refrigerating and Air-Conditioning Engineers (ASHRAE) 2014; Standard 55-2013. https://www.ashrae.org/standards-research--technology/standards--guidelines.

[16] Shrestha I., Shrestha S. (2005). Indoor Air Pollution from Biomass Fuels and Respiratory Health of the Exposed Population in Nepalese Households. Int. J. Occup. Env. Health.

[17] Mengersen K., Morwaska L., Wang H., Murphy N., Tayphasavanh F., Darasavong K., Holmes N. (2011). The effect of housing characteristics and occupant activities on the respiratory health of women and children in Lao PDR. Sci. Total Environ..

[18] Siddiqui A., Lee K., Gold E., Bhutta Z. (2005). Eye and respiratory symptoms among women exposed to wood smoke emitted from indoor cooking: A study from southern Pakistan. Energy Sustain. Dev..

[19] Zhang J., Smith K. (2007). Household air pollution from coal and biomass fuels in China: Measurements, health impacts, and interventions. Environ. Health Perspect..

[20] Ansari F., Khan A., Patel D., Siddiqui H., Sharma S., Ashquin M., Ahmad I. (2010). Indoor exposure to respirable particulate matter and particulate-phase PAHs in rural homes in North India. Environ. Monit. Assess..

[21] Saksena A., Prasad R., Pal R., Joshi V. (1992). Patterns of daily exposure to TSP and CO in the Gatwal Himalaya. Atmos. Environ..

[22] Smith K., Aggarwal A., Dave R. (1983). Air pollution and rural biomass fuels in developing countries: A pilot village study in India and implications for research and policy. Atmos. Environ..

[23] Ingale L., Kamalesh D., Dhananjay S., Attarde S., Ingle S. (2011). Monitoring and respiratory health assessment of the population exposed to cooking fuel emissions in a rural area of Jalgaon district, India. Asia-Pac. J. Public Health.

[24] Balakrishnan K., Parikh J., Sankar S., Padmavathi R., Srividya K., Venugopal V., Prasad S., Pandey V. (2002). Daily Average exposures to respirable particulate matter from combustion of biomass fuels in rural households of southern India. Environ. Health Perspect..

[25] Colbeck I., Nasir Z., Hasnain S., Sultan S. (2008). Indoor air quality at rural and urban sites in Pakistan. Water Air Soil Pollut. Focus.

[26] Park E., Lee K. (2003). Particulate exposure and size distribution from wood burning stoves in Costa Rica. Indoor Air.

[27] Boleij J., Ruigewaard P., Hoek F. (1989). Domestic air pollution from biomass burning in Kenya. Atmos. Environ..

[28] Bruce N., Perez-Padilla R., Albalak R. (2000). Indoor air pollution in developing countries: A major environmental and public health challenge. Bull. World Health Organ..

